# Deciding when to move

**DOI:** 10.7554/eLife.85477

**Published:** 2023-01-31

**Authors:** Lauren Sullivan

**Affiliations:** 1 Department of Plant Biology, Michigan State University East Lansing United States; 2 Ecology, Evolution, and Behavior Program, Michigan State University East Lansing United States; 3 W. K. Kellogg Biological Station, Michigan State University Hickory Corners United States

**Keywords:** taraxacum officinale, Dandelion, seed dispersal, plant aerodynamics, biomechanics, mechanical ecology, movement ecology, Other

## Abstract

Dandelion seeds respond to wet weather by closing their plumes, which reduces dispersal when wind conditions are poor.

**Related research article** Seale M, Zhdanov O, Soons MB, Cummins C, Kroll E, Blatt MR, Zare-Behtash H, Busse A, Mastropaolo E, Bullock JM, Viola IM, Nakayama N. 2022. Environmental morphing enables informed dispersal of the dandelion diaspore. *eLife* **11**:e81962. doi: 10.7554/eLife.81962.

When you think about plants spreading their seeds, you may conjure an image of dandelion seeds being carried away by the wind. In fact, if you have ever blown on a dandelion to make a wish, you may have helped a plant disperse its seeds to new habitats where it can reproduce more easily. Understanding how far seeds travel is important for predicting whether plant species will be able to keep pace with climate change and migrate to places with the right conditions for them to continue to grow (Corlett and Westcott, 2013).

As plants cannot move themselves to a new location, they rely on vectors to disperse their seeds away from them (Clobert et al., 2012). Some plants depend on non-living parts of an ecosystem to move their seeds: the dandelion, for instance, depends on the wind while seeds of other species may be carried by flowing water. Plants can also exploit the active movement of animals. For example, seeds can be consumed by animals or stuck to their fur or feathers, and thus be dispersed over large distances as animals often migrate when foraging for food or searching for a mate (Vittoz and Engler, 2007).

It is largely believed that plants that rely on non-living mechanisms are mostly passive in how their seeds are dispersed and have no control over where their seeds will eventually land. Now, in eLife, Naomi Nakayama and colleagues – including Madeleine Seale as first author – report new findings that turn this assumption on its head (Seale et al., 2022a). The team (who are based the University of Edinburgh and various institutes in the United Kingdom and The Netherlands) found that dandelion seeds can sense the environment and modify their shape to alter their own dispersal depending on the weather.

To understand how dandelion seeds move under both dry and wet conditions, Seale et al. placed individual seeds in a wind tunnel at different levels of humidity. In agreement with previous studies, they found that the hairs dandelion seeds use to travel via the wind are more spread out in dry environments, resulting in an open plume structure (Figure 1A). This configuration creates an air vortex ring above the plume that allows the seed to remain in the air for longer periods of time (Cummins et al., 2018). However, under humid conditions, such as in wet weather, the plume structure closes due to the swelling of cells on the flowering portion of the dandelion, also known as the seed head (Seale et al., 2022b; Figure 1B). The wind tunnel experiments by Seale et al. showed that when the seed has a closed structure, the vortex ring shrinks and moves closer to the plume. This changes the aerodynamics of the seed and causes it to fall faster, thus limiting how far it can travel by wind (Figure 1A and B).

**Figure 1.**
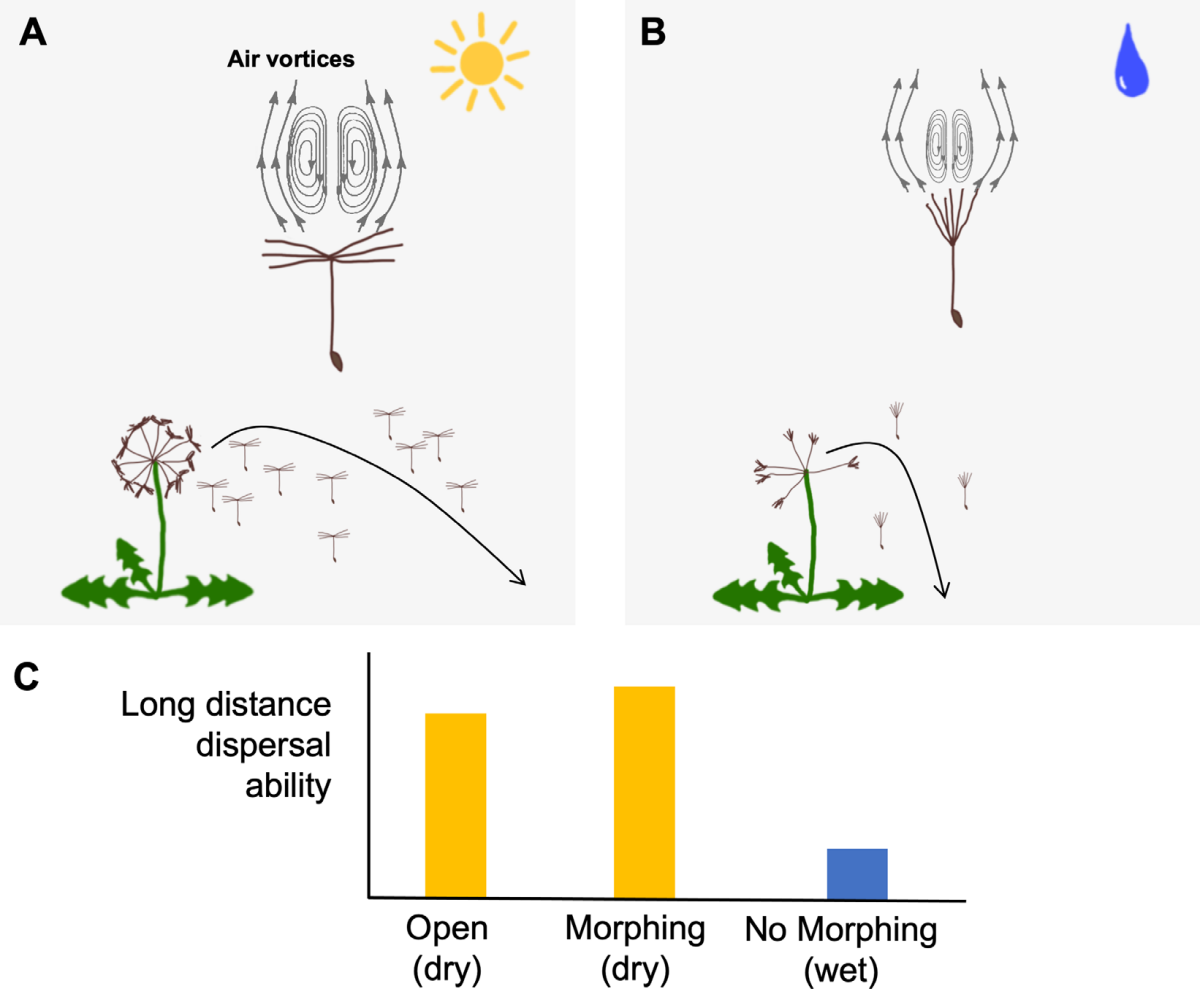
Dandelion seeds change their shape in wet conditions, which alters their overall movement. (**A**) Dandelion seeds can travel longer distances under dry conditions by opening their plumes. This configuration creates large vortices of air (represented by arrows) that help the seed stay aloft for longer, allowing it to travel further away from the plant. (**B**) Under wet conditions seeds change their shape to a closed state which only generates small vortices of air that cannot move seeds as far. However, the closed shape makes it harder for seeds to detach from the head of the dandelion, increasing the chance that seeds only disperse when conditions are more favorable (e.g., when it is dryer and windier). (**C**) The ability of seeds to morph (change shape between closed and open) leads to a larger proportion moving long distances under dry conditions compared to seeds that are always open (left yellow bar) and seeds that cannot morph under wet conditions (blue bar).

Seale et al. then investigated how humid conditions altered the chance that a seed would be released by placing whole seed heads in the wind tunnel. This revealed that those with a closed plume structure were much less likely to detach under wet conditions. In contrast, when conditions were dry, more dandelion seeds with open plumes dislodged from the seed head, particularly at high horizontal wind speeds which have been shown to increase seed detachment (Greene, 2005). These findings suggest that under humid, wet conditions (when wind speeds are typically lower) seed dispersal is initiated much less frequently. Seeds are then more likely to detach under dry conditions, which according to previous work leads to wind updrafts that pull the seeds high into the atmosphere, causing them to disperse over longer distances (Tackenberg et al., 2003).

Finally, Seale et al. explored how their wind tunnel results scaled up to affect how dandelion seeds disperse in the real world. The team used a simulation model that accounts for realistic wind, temperature and humidity dynamics (Soons et al., 2004) incorporated from local sources in Scotland. They found that under dry conditions, a greater percentage of seeds that were able to morph between open and closed plumes traveled longer distances than seeds that remained open (Figure 1C). In addition, if seeds had a closed plume, they were more likely to remain on the plant when conditions were not good for movement, i.e. when wind speed is low and/or there is high humidity.

The results of this study suggest that seeds can, in fact, sense their environment and that this environmental sensing allows plants to maximize their dispersal under variable conditions. This interdisciplinary work serves as a model for answering questions related to passive seed dispersal and showcases the power in connecting cellular mechanisms to aerodynamics and the ecological consequences of dispersal. Further studies could address whether other plant species can sense the humidity of their environment and morph accordingly so they only spread when weather conditions are best for moving long distances. This could influence their ability to adapt to changing wind patterns and increasingly warming temperatures caused by climate change (Kling and Ackerly, 2020).

## References

[bib1] Clobert J, Baguette M, Benton TG, Bullock JM (2012). Dispersal Ecology and Evolution.

[bib2] Corlett RT, Westcott DA (2013). Will plant movements keep up with climate change?. Trends in Ecology & Evolution.

[bib3] Cummins C, Seale M, Macente A, Certini D, Mastropaolo E, Viola IM, Nakayama N (2018). A separated vortex ring underlies the flight of the dandelion. Nature.

[bib4] Greene DF (2005). The role of abscission in long-distance seed dispersal by the wind. Ecology.

[bib5] Kling MM, Ackerly DD (2020). Global wind patterns and the vulnerability of wind-dispersed species to climate change. Nature Climate Change.

[bib6] Seale M, Zhdanov O, Soons MB, Cummins C, Kroll E, Blatt MR, Zare-Behtash H, Busse A, Mastropaolo E, Bullock JM, Viola IM, Nakayama N (2022a). Environmental morphing enables informed dispersal of the dandelion diaspore. eLife.

[bib7] Seale M, Kiss A, Bovio S, Viola IM, Mastropaolo E, Boudaoud A, Nakayama N (2022b). Dandelion pappus morphing is actuated by radially patterned material swelling. Nature Communications.

[bib8] Soons MB, Heil GW, Nathan R, Katul GG (2004). Determinants of long-distance seed dispersal by wind in grasslands. Ecology.

[bib9] Tackenberg O, Poschlod P, Kahmen S (2003). Dandelion seed dispersal: the horizontal wind speed does not matter for long-distance dispersal - it is updraft!. Plant Biology.

[bib10] Vittoz P, Engler R (2007). Seed dispersal distances: a typology based on dispersal modes and plant traits. Botanica Helvetica.

